# Psychedelic therapy: bridging neuroplasticity, phenomenology, and clinical outcomes

**DOI:** 10.3389/fpsyt.2025.1637162

**Published:** 2025-09-01

**Authors:** Ronit Kishon, Yael M. Cycowicz

**Affiliations:** ^1^ New York State Psychiatric Institute (NYSPI), New York, NY, United States; ^2^ Department of Psychiatry, Columbia University Irving Medical Center, New York, NY, United States

**Keywords:** psychedelics, psychedelic therapy, precision medicine, precision psychiatry, precision psychotherapy, neuroplasticity, depression

## Abstract

The resurgence of interest in psychedelic compounds as potential treatments for psychiatric disorders represents a paradigm shift in mental health care. Psychedelics exert their effects through serotonergic modulation, particularly via 5-HT2A receptor activation, inducing profound alterations in consciousness and catalyzing neuroplasticity. While their neurobiological effects are well-documented, the therapeutic potential of psychedelics extends beyond their biochemical properties. Psychedelic therapy provides a structured framework for integrating the psychedelic experiences, comprising three key phases: preparation, administration, and integration. While current clinical trials focus primarily on establishing pharmacological efficacy across diagnostic categories, the phenomenology of psychedelic experiences offers valuable insights into precision psychiatry. Emerging evidence suggests that therapeutic benefits arise not only from acute symptom relief but also from enduring changes in self-perception, emotion regulation, and interpersonal connectedness. This paper explores the psychological dimensions of psychedelic therapy, emphasizing its implications for psychiatric treatment. By integrating neuroscientific findings with phenomenological insights, we argue that psychedelics represent more than a novel pharmacological intervention. They offer a fundamentally different therapeutic model that necessitates a reconceptualization of mental health treatment. Further research is required to refine treatment protocols, elucidate the relationship between subjective experiences and therapeutic outcomes, and establish best practices for integrating psychedelics into clinical settings.

Improving mental health and addressing the challenges of inadequate treatment for severe mood disorders have prompted clinicians and researchers to re-examine the potential of psychedelic drugs for treating various disorders including depression, anxiety, PTSD, and substance use (1–3). Psychedelics, including psilocybin, LSD (lysergic acid diethylamide), DMT (dimethyltryptamine), mescaline, and atypical psychedelic ibogaine, are psychoplastogenic class of drugs capable of rapidly inducing neural plasticity and therefore are seen as a paradigm shift in psychiatric treatment approach (4–7). Other drugs that are “psychedelic-like,” such as Ketamine and MDMA have also shown promising potential in treating a variety of psychiatric disorders (8, 9).

The term “psychedelic” originates from the Greek words psychē (ψυχή), meaning “soul,” and dēloun (δηλoῦν), meaning “to make visible” or “to reveal,” signifying the mind-revealing nature of these substances. Ingestion of psychedelic drugs induces altered states of consciousness that engage various sensory and cognitive modalities, often leading to deeply personal recollections intertwined with an individual’s life experiences. These experiences, while sometimes painful or anxiety provoking, are typically transient and can be profoundly meaningful. Some patients report transcendental or spiritual states (10–15), which can foster a more receptive and contemplative approach to their difficult emotions and self-perception. While the neurobiological mechanisms underlying these experiences are likely universal, their subjective experiences vary across individuals, highlighting the potential for personalized therapeutic approaches. Current clinical trials primarily aim to establish the efficacy of various psychedelic substances across a wide range of diagnostic categories, which differ significantly. As psychoactive substances, psychedelics produce profound alterations in a state of consciousness and cause changes in perception, mood, thought processes, and positive affect (16, 17), persisting for months even after a single dose (7, 18–21). We argue that the unique phenomenological experiences elicited by psychedelics provide invaluable insights into an individual’s inner world, which could fundamentally advance the goals of precision psychiatry. In this context, precision psychiatry seeks to tailor treatment by aligning specific interventions with patients’ biological, psychological, and contextual profiles. This approach integrates advances in neuroscience, trauma research, personality theory, and cultural psychiatry to move beyond one-size-fits-all models.

Psychedelics exert their hallucinogenic effects primarily through their interaction with serotonin receptors, particularly the 5-HT2A receptor type, in the brain. The structural and functional neuroplasticity that occurs within a few hours post-administration is likely due to gene expression changes (22–24), promoting a significant increase in dendrite and spine formation (25, 26). In animal models, these effects peak within a few days and tapper off over a week, although some of the structural and functional transformation may persist for weeks (25). Neuroimaging studies in humans have shown changes in functional connectivity shortly after administration of various psychedelic compounds (27–33). However, fewer studies, and those that exist are less consistent, demonstrating long-term changes in brain connectivity when the neuroimaging examination occurred days or weeks after dosing sessions (34, 35). Nevertheless, proponents of psychedelics for psychiatric treatment theorize that enduring neural changes can improve outcomes for patients with mood disorders (36).

The relationship between psychedelic drug effects and psychological therapeutic outcomes appears to involve multiple levels of action. To explain psychedelics’ therapeutic effects, researchers have proposed two main mechanisms: either through dissolving rigid cognitive patterns and enabling integration of new insights via increased neuroplasticity (37), or by temporarily reopening developmental-like periods of heightened brain malleability that enhance responsiveness to therapeutic intervention (38). However, further research is needed to validate these proposed mechanisms of action.

It’s important to recognize that the effectiveness of psychedelic treatment arises from a complex interplay between the drug’s biochemical effects (such as the type of substance and dosage) and non-pharmacological factors, including contextual and environmental influences (39, 40). The safety, clinical benefits, and therapeutic outcomes of psychedelic interventions are widely considered to be highly dependent on such non-drug factors, with ‘set’ and ‘setting’ playing essential roles (41–43). The concept of ‘set and setting’ was introduced by Harvard psychologist Timothy Leary (44). According to Leary’s hypothesis, psychedelic drugs function as a magnifying glass for consciousness, intensifying whatever is present in an individual’s mind. He defined ‘set’ as encompassing one’s personality, the effects of preparation for the drug on one’s mind, and one’s expectations and intentions before receiving the drug. ‘Setting,’ on the other hand, refers to the environmental factors, including the physical, social, and cultural contexts in which the experience takes place. The multifactorial nature of psychedelic experiences arising from the interplay of neurobiological mechanisms, individual psychology, and environmental factors demands an integrative framework for both research and clinical implementation (40, 45). Current studies vary widely in design and are often limited by small sample sizes, short follow-up periods, and challenges in maintaining blinding (12, 46, 47). While the drug dosage, context, and the environment in which psychedelics are administered are meticulously monitored and controlled in clinical trials (46), their precise influence on behavioral outcomes, as well as the role of cognitive and psychological processes and their therapeutic potential, remain unclear and require further study.

Historically, psychedelic therapeutic models have varied in their intervention level, ranging from active psychotherapeutic engagement following low-dose administration to minimal psychological support during high-dose sessions, where participants navigate their experience independently (48). While current clinical trials tend to favor the latter approach, research indicates that optimal therapeutic outcomes critically depend on ensuring both the safety and meaningfulness of the experience (49). Given the strong influence of suggestibility and expectancy on therapeutic outcomes, proper guidance is crucial (50, 51). Studies suggest that patients experience poorer treatment outcomes when they either avoid their experiences or remain in prolonged states of heightened physiological and emotional arousal (52).

While psychedelic therapy holds promise, it also carries significant risks that require careful evaluation. Acute effects may include psychotic-like symptoms, loss of control, and resurfacing of traumatic memories, while prolonged reactions can involve anxiety, dissociation, or trauma-like symptoms, including rare cases of hallucinogen persisting perception disorder (HPPD) (53). These risks emphasize the importance of rigorous patient screening and careful selection procedures (54). Furthermore, these potential adverse events highlight the crucial role of a trained, supportive, and compassionate therapist (55, 56). A strong therapeutic alliance, grounded in trust and safety, is essential for positive outcomes (49, 57). Because therapists often interact with patients only a few times throughout the psychedelic administration (i.e., before, during, and after the session), they must embody flexibility, honesty, warmth, and respect, to facilitate positive outcomes (58). The therapist’s role is not to direct but to support the patient through the process (54). By fostering a safe, non-directive environment that encourages exploration while honoring the patient’s autonomy, therapists can help maximize the benefits of the psychedelic experience and minimize risks of undue influence or suggestion, ultimately promoting lasting and meaningful change.

This understanding has led to the development of Psychedelic Assisted Therapy (PAT), a structured psychological support framework implemented in most clinical studies. PAT integrated insights from historical psychedelic research, clinical applications, contemporary neuroscience findings, and principles from evidence-based psychotherapies (59–61). It facilitated profound psychological insights and perceptual shifts that are correlated with symptom reduction, as demonstrated by clinical trials (2, 20). Building on these theoretical insights, PAT emphasized a supportive therapeutic environment encompassing thorough preparation, careful monitoring during drug administration, and post-experience integration (62). Three fundamental principles were the basis of PAT: establishing a secure emotional environment, maintaining awareness of the present experience, and empowering participants’ autonomy. Each of these principles was designed to optimize therapeutic outcomes and facilitate the integration of psychedelic experiences (62). While the term PAT has been widely used in early clinical trials to describe structured psychotherapeutic protocols surrounding psychedelic dosing (e.g., MAPS Phase 2, COMP001), the field is increasingly shifting toward more inclusive terms such as ‘psychedelic therapy’ or ‘psychedelic treatment’. These terms reflect a broader therapeutic model in which the full psychedelic experience, including both the drug and its surroundings, is considered as central to clinical change. However, determining the optimal method of support remains an important area for further research.

An alternative, more neutral framework is the psychedelic education model, which has emerged in recent clinical trials (54). This model seeks to minimize subjective psychological influence and isolate the pharmacological effects of the drug. While ethically grounded in ensuring safety, it reflects a minimalistic view on the role of psychological support, arguably offering a more neutral approach than the relational framework used in PAT. In the psychedelic education model, the professional guiding the patient is typically referred to as a *monitor* rather than a therapist, reflecting a non-directive role that emphasizes safety and support over delivering psychotherapeutic intervention. Although both PAT and psychedelic education models aim to foster a safe environment and reduce experiential avoidance, their underlying philosophies and methods differ significantly.

According to PAT, in the preparatory phase, therapists work with patients to explore their background, help them understand their symptoms and treatment goals, and establish a strong therapeutic rapport (46). This phase also includes psychoeducation, equipping patients with an understanding of the range of experiences they may encounter under the influence of psychedelics and strategies for navigating potential challenges (47). A key skill developed during preparation is the patient’s ability to engage with different mental states in a balanced way, neither suppressing nor being overwhelmed by them, while ensuring a calm and controlled therapeutic setting. To support this, therapists guide patients through experiential exercises that can be used during the dosing session. Given that physical support or reassurance may sometimes be necessary during the psychedelic session, therapists establish clear protocols and boundaries regarding appropriate physical contact before beginning treatment (63). Since psychedelic experiences are inherently unpredictable, a crucial aspect of preparation is helping patients set intentions rather than expectations for the experience and its outcomes. Patients are encouraged to understand that both the immediate effects and the long-term changes cannot be fully anticipated or controlled. Setting an intention provides a flexible psychological framework, allowing patients to process whatever emerges during the session (64). Additionally, patients are guided to remain open to all aspects of the psychedelic experience, including challenging or distressing feelings, with the understanding that these experiences are transient (62). In contrast, the psychedelic education model limits the preparation to providing factual information regarding potential physical and psychological effects without delving into the participant’s emotional history, mental health symptoms, or treatment goals. While monitors may address basic concerns or fears, they do not engage in psychotherapeutic dialogue. As we highlight the benefits of structured therapeutic frameworks such as PAT, it is important to acknowledge that some clinical trials have intentionally adopted more minimalist models such as the psychedelic education approach. These methods are often motivated by practical considerations, including regulatory constraints, scalability across diverse clinical settings, and efforts to reduce therapeutic imprinting or bias. In certain cultural or health system contexts, less intensive models may be both more feasible to implement and more acceptable to participants. A nuanced understanding of these factors is essential as the field moves toward broader real-world application. Future research should explore how varying levels of psychotherapeutic support influence both accessibility and clinical outcomes.

The drug administration phase typically lasts between 3 to 8 hours, depending on the type of psychedelic used, during which a moderate to high dose is administered under the continuous supervision of a therapist/monitor who remains with the patient until the acute effects subside. Patients are generally placed in a comfortable, non-clinical environment, where they lie down on a bed or sofa. In PAT, after ingesting the psychedelic, they are encouraged to focus inward, often with their choice of music and the use of eye shades (2, 65). During this 3–8 hour period, the therapist’s primary role is to provide a supportive presence, fostering a sense of safety and openness while allowing the patient’s experience to unfold naturally under the influence of the substance (65, 66). The therapist does not direct or interfere with the experience but remains available to offer reassurance if needed. By comparison, the psychedelic education model positions the monitor in a neutral and supervisory role, focused primarily on ensuring safety, while intentionally avoiding deeper emotional or psychotherapeutic engagement. This approach deliberately limits the relational depth to isolate pharmacological effects, reflecting a fundamentally different therapeutic philosophy.

In the final integration phase of the PAT, which often consists of 2–3 sessions, therapists assist patients in processing and interpreting their psychedelic experience. The primary goal is to help patients translate their insights into meaningful, long-term changes in their lives (49, 67). Rather than providing interpretations or employing structured therapeutic techniques, therapists guide patients in integrating their own physical, mental, and emotional/spiritual experiences, including any insights gained (49). This phase is optional and limited in the psychedelic education model. When provided, the monitor may conduct a brief debriefing with the patient, acknowledging their experience without exploring emotional themes or linking insights to clinical symptoms.

While debate continues over whether the acute psychedelic experience is necessary for therapeutic benefit, and some researchers advocate for non-hallucinatory psychedelics that eliminate it (68), clinical observations provide growing evidence suggesting that the quality and nature of the psychedelic experience itself critically predict long-term outcomes (54). Psychedelics offer access to multiple domains of psychological functioning, including autobiographical memory, emotional processing, body awareness, and verbalization (69). This multidimensional window into the patient’s inner world, occurring during and after the psychedelic experience reveals insights often inaccessible through traditional psychiatric or psychological treatments, illuminating how individuals process emotion, construct meaning, and integrate experience. During these states, patients frequently confront unconscious material and unresolved trauma, resulting in emotional experiences that can be cathartic and healing, yet at times disorienting and anxiety-provoking (70). Therefore, therapeutic value arises not only from the acute psychedelic state but also from the integration phase, during which sensory, cognitive, and emotional material is processed and transformed into lasting behavioral change. Integration is especially crucial for meaning-making and anchoring insights. While current PAT protocols typically include two to three integration sessions, additional sessions may be beneficial during this phase. Given that emotional and cognitive systems often remain more flexible and receptive following the psychedelic experience, extending integration sessions could significantly enhance the depth and long-term durability of therapeutic outcomes.

Research shows that psychedelic therapy often enhances self-compassion while reducing shame, self-criticism, and blame (71, 72). Moreover, mystical-type experiences, which are characterized by unity, sacredness, transcendence, and ineffability, are associated with greater acute symptom improvement (73, 74). These qualitative patterns are supported by quantitative data demonstrating increases in self-compassion and improved emotional regulation following psychedelic treatment (12, 13, 41). Using qualitative methods, these psychological shifts have been categorized into overlapping domains: enhanced self-awareness and self-understanding; altered self-perception including increased self-efficacy and reduced self-criticism; heightened connection both internally (with emotions and identity) and externally (with others and the world); transcendent experiences; and an expanded emotional range encompassing love, joy, sadness, and anger (9). These emotional domains appear consistently across different psychedelics and diagnostic groups, suggesting fundamental mechanisms of change (14, 15). However, psychedelic experiences vary widely across individuals. We propose that these experiences may serve both diagnostic and therapeutic functions, offering valuable insight to guide subsequent care. Clinical observations made during psychedelic sessions, combined with systematic documentation of individual experiences, have the potential to enhance psychiatric care by supporting individualized treatment planning. For example, patients whose psychedelic experiences are primarily somatic may benefit from different follow-up strategies than those whose experiences are more autobiographical or emotionally centered. Stratifying patients based on experiential patterns may help identify subgroups with distinct therapeutic needs (75), advancing a model that integrates precision medicine with patient-centered care. Self-report measures such as the Mystical Experience questionnaire (MEQ) (73), Ego Dissolution Inventory (EDI) (76), and the quantification of recorded narratives offer preliminary tools for systematically classifying psychedelic experiences. Future research should aim to link these experiential profiles to treatment outcomes, thereby refining approaches to patient stratification and ultimately guiding the development of optimal individualized treatment.

Psychological and physiological variables measured after psychedelic administration may correlate with subjective experiences, offering insights into how to better match patients with specific interventions. For instance, compared to patients with social-personal experiences during dosing, those who demonstrate predominantly somatic experiences may show different integration needs or psychotherapeutic support. Detailed case-level analyses can further inform psychotherapy protocols and guide adjunctive pharmacological treatments in the weeks and months following dosing (64). This approach aligns with the principles of precision psychiatry by integrating patients’ psychological profiles and neurobiological measures to tailor post-treatment care. While additional research is needed to standardize assessments and validate outcomes, this therapeutic model promotes a scientifically rigorous framework that values both objective and subjective experiences. Advancing this framework will require studies aimed at refining screening methods, enhancing therapeutic support, and optimizing post-psychedelic treatment care. Although broad therapeutic processes have been identified (9, 71, 72), more systematic analysis could improve patient selection and aftercare. Moving beyond exclusionary criteria toward personalized support models represents a critical step in realizing the full potential of psychedelic-assisted therapy within precision psychiatry.

Psychedelic therapy represents a transformative shift in the treatment of mental health disorders by uniting biological mechanisms with profound psychological exploration. Through the promotion of neural plasticity, psychedelics open a window of heightened brain adaptability, creating opportunities to access and engage with core psychological processes. This unique interplay offers a powerful foundation for advancing personalized, precision-based mental health care. With careful documentation and ongoing refinement of clinical protocols, psychedelic therapy holds promise for advancing a more effective, nuanced, and person-centered model of psychiatry.
